# Laparoscopic-assisted versus ultrasound-guided transversus abdominis plane block for laparoscopic cholecystectomy: a systematic review and meta-analysis

**DOI:** 10.1186/s12893-024-02706-7

**Published:** 2024-12-21

**Authors:** Sundus Abdul Ghani, Hassan Ul Hussain, Maryam Abdul Wahid, Neha Majeed, Sheeba Burney, Areesha Tanveer, Muhammad Sohaib Asghar

**Affiliations:** 1 Dow University of Health Sciences, Karachi, Pakistan; 2 Mayo Clinic, Rochester, MN, USA; 3 AdventHealth Sebring, FL, USA

**Keywords:** Cholecystectomy, Ultrasound-guided, Laparoscopic, Tranversus abdominis plane, Anesthesia

## Abstract

**Background:**

Laparoscopic-assisted (LTAP) and ultrasound-guided (UTAP) transversus abdominis plane (TAP) blocks are widely used for postoperative analgesia in laparoscopic cholecystectomy (LC), yet their comparative effectiveness remains unclear. The aim of this meta-analysis was to systematically evaluate and compare postoperative outcomes of LTAP and UTAP in LC.

**Materials and methodology:**

A comprehensive literature search of five electronic databases was conducted from the inception of the paper till 2 June 2024 following PRISMA guidelines. Eligibility criteria included: (a) randomized controlled trials (RCTs); (b) adult patients (≥ 18 years) undergoing elective LC; (c) intervention group undergoing LTAP; (d) control group receiving UTAP; (e) outcomes: postoperative pain intensity using VAS score; time to first analgesic need; postoperative morphine consumption; postoperative nausea vomiting (PONV); time to first bowel evacuation; time to first flatus. Mendeley Desktop 1.19.8 was used for article retrieval and for the removal of duplicates. Risk of bias was assessed using the Cochrane Risk of Bias Tool, and statistical analysis was performed using Review Manager, applying a random-effects model. Forest plots represented combined effects of Risk Ratios (RRs) for dichotomous outcomes and weighted mean differences (WMDs) for continuous outcomes with a 95% confidence interval (CI). P-value ≤ 0.05 was considered statistically significant and Higgin’s I² test was employed to assess heterogeneity.

**Results:**

Seven RCTs in total involving 603 patients were included in the analysis, with 236 patients in the LTAP group and 232 in the UTAP group. No statistically significant differences observed between LTAP and UTAP in postoperative pain intensity at 6, 12, and 24 h, time to first analgesic need, postoperative morphine consumption, PONV, time to first stools, and time to first flatus, initially. Sensitivity analysis revealed a significant reduction in 6-hour postoperative pain in the LTAP group (WMD = 0.39; 95% CI = 0.10,0.67; *P* = 0.008; I² = 0%), but no significant differences were found in later time points (12 h: WMD = 0.12; 95% CI = -0.17,0.40; *P* = 0.42; I² = 0%; 24 h: WMD = -0.04; 95% CI = -0.26, 0.18; *P* = 0.73; I² = 5%) or in other outcomes. Moderate levels of heterogeneity and an overall low risk of bias in quality assessment were observed among the studies.

**Conclusion:**

Our meta-analysis indicated no clear advantage of LTAP over UTAP in managing postoperative pain and related outcomes in LC. Although LTAP may offer logistical benefits by reducing the need for equipment and personnel, further large-scale RCTs focusing on procedure-specific outcomes are needed to establish definitive conclusions.

**Supplementary Information:**

The online version contains supplementary material available at 10.1186/s12893-024-02706-7.

## Introduction

Laparoscopic surgery (LS) has revolutionized the practice of general surgery by cutting down on surgical complications like duration of hospital stay, wound infection rates, and postoperative pain. LSs are performed via smaller incisions that yield patients’ early mobilization and reduce perioperative morbidity [1]. Despite these outstanding advantages, laparoscopic procedures result in unexpectedly high-intensity postoperative pains [2]. Laparoscopic interventions are considered the gold standard procedure for cholecystectomies. They are indicated mainly in patients with complicated gallbladder stones and gallbladder polyps [3]. 

In laparoscopic cholecystectomy (LC), postoperative pain leads to significant morbidity and demands effective management [4]. Incision wounds on the anterior abdominal wall are painful and challenging to resolve [3]. There are several methods for pain control in LC including nonsteroidal anti-inflammatory drugs (NSAIDs), injecting the intraperitoneal cavity with local anesthetic or normal saline, low-pressure gas, and nitrous oxide pneumo-peritoneum [5]. Similarly, opioids for postoperative pain relief have been extensively used in the past. However, chronic use of opioids comes with a long list of drawbacks including misuse, abuse, and addiction [6]. 

The increasing interest in using multimodal analgesic techniques that suggest a combination of opioids, NSAIDs, and local anesthetics for effective pain management [7] brings attention to one such component of multimodal analgesic therapy, the transversus abdominis plane (TAP) block. TAP was first described by Rafi in 2001 [8]. It is a technique of infiltrating local anesthesia in between the neurovascular plane of muscles internal oblique and transverse abdominis of the abdominal wall [9]. TAP block halts sensory nerve afferents carrying information from thoracic intercostal nerves 7–12, ilioinguinal nerve, iliohypogastric nerve, and lumbar nerves 1–3 in the lateral cutaneous branches. These neural afferents are approached via ilio-lumbar triangles of Petit [9]. 

TAP block is usually performed by visualization of the triangle of Petit under ultrasound. The ultrasound probe is placed on the lateral abdominal wall along the anterior axillary line and halfway between the costal margin and iliac crest. This technique of TAP block administration is well-known due to its reliability and safety profile [10]. However, an alternate method of administering TAP block has surfaced, laparoscopic-assisted TAP block (LTAP). This technique involves creating a pneumoperitoneum and visualizing the internal abdominal wall through a laparoscopic camera. While injecting the local anesthetic, the internal bulge of the abdominal wall confirms the presence of injection in the abdominal wall. This minimizes the need for additional medical equipment inside the theater [11]. 

Ample literature in the form of randomized controlled trials (RCTs) comparing the two procedures of TAP block is available. However, the lack of a systematic analysis and meta-analysis that gathers data from multiple studies held in different times and regions over the globe warrants a comprehensive paper. Our study, being the first of its kind, provides valuable pooled results by encompassing outcomes of all the present RCTs.

The objective of this systematic review and meta-analysis is to compare the safety and efficacy of two techniques of TAP block, Ultrasound-guided transversus abdominis plane (UTAP) and LTAP in patients undergoing LC. We aim to analyze post-operative pain intensity via visual analog scale (VAS) score, time to first analgesic need, time to first bowel evacuation, and time to first flatus.

## Materials and methodology

Our meta-analysis was conducted in accordance with the Preferred Reporting Items for Systematic Review and Meta-Analysis (PRISMA) guidelines and followed the Cochrane collaboration framework [12]. This systematic review and meta-analysis has been registered on Prospero (ID: CRD42024551599). The PRISMA checklist for this study is provided in Supplementary Table 1.

### Data sources and search strategy

For this meta-analysis, a systematic search of the literature, comparing the effectiveness of LTAP and UTAP for laparoscopic cholecystectomy was conducted across five electronic databases (PubMed, Google Scholar, Cochrane Library, ScienceDirect, and ClinicalTrials.gov), from inception till 2 June 2024. Our search string included the integration of the following Medical Subject Headings (MESH terms) along with necessary Boolean operators: “Laparoscopic”; “laparoscopy”; “assisted”; “guided”; “supported”; “aided”; “ultrasound”; “ultrasonography”; “US”; “sonar”; “ultrasonic”; “sonographic”; “TAP”; “transversus abdominis plane block”; “cholecystectomy”. No restrictions regarding time, language, or location were imposed during the literature search. Gray and white literature searches were performed to ensure that no study goes unnoticed. Further information regarding the detailed search strategy used in each database is available in Supplementary Table 2.

### Study selection and eligibility criteria

The articles retrieved from the comprehensive literature search were imported into Mendeley Desktop 1.19.8 (Mendeley Ltd., Amsterdam, Netherlands), where duplicates were identified and eliminated. Two independent reviewers (MAW, SAG) thoroughly assessed the remaining articles to ensure adherence to predefined PICO criteria. Any discrepancies were resolved by a third author (HUH). Initially, the papers were selected based on their abstracts and were later evaluated comprehensively based on their full texts. This study exclusively included randomized controlled trials (RCTs) that assessed the effectiveness of LTAP versus UTAP in adults (aged 18 years and older) undergoing elective LC. The intervention group received the LTAP block while the control group received the UTAP block. The outcomes of interest included postoperative pain intensity using the VAS score, time to first analgesic need, postoperative morphine consumption, postoperative nausea vomiting (PONV), time to first bowel evacuation, and time to first flatus. Articles published in languages other than English, studies involving pediatric patients, studies not categorized as RCTs, studies lacking full-text availability, case reports, meta-analyses, narrative and systematic reviews were excluded from our paper.

### Data extraction

Data on study and population characteristics were extracted and incorporated into a Microsoft Excel sheet. It included the first author’s name, publication year of study, study design, region of study, sample size, mean age, number of males and females, mean body mass index (BMI), mean operation time, number of patients undergoing intervention and control groups, and primary and secondary outcomes. Data extraction was conducted by two researchers independently (SAG and SB) which was independently reviewed by a third author (HUH).

### Risk of bias and quality assessment

Two reviewers (SAG and SB) independently assessed the quality of the seven included RCTs. A third independent reviewer (HUH) was consulted to address any differences in the risk of bias evaluations between the two reviewers. We used the Cochrane Risk of Bias Tool [13] to assess the quality of included studies, categorizing the risk of bias as either high, low, or unclear. RCTs were assessed across five domains including (i) randomization procedure; (ii) variations from proposed interventions; (iii) incomplete outcome data; (iv) outcome assessment; (v) choice of reported results.

### Statistical analysis

Two authors (NM and MAW) performed the statistical analysis using Review Manager (version 5.4.1). The results were depicted through the forest plots that represent the combined effect of Risk Ratios (RRs) for dichotomous outcomes and weighted mean differences (WMDs) with a 95% confidence interval (CI). Inverse variance and Mantel-Haenszel statistical methods were used for continuous and dichotomous outcomes, respectively while applying a random-effects model to ensure the accuracy of the results. Medians and Interquartile ranges (IQRs) were converted to mean and standard deviation using VassarStats, an online calculator [14]. 

The threshold for statistical significance was defined by a p-value less than 0.05 and with a CI of 95% to assess the implications of these findings. Higgin’s I² test was also used to evaluate the degree of heterogeneity, categorized mild (25–50%), moderate (50-75%) or high (> 75%) [15]. 

Sensitivity analysis was performed individually by leave-one-out method in cases of moderate (50–75%) and high heterogeneity (> 75%). This enabled us to determine which individual studies were leading to high heterogeneity.

## Results

### Study selection

**Fig. 1:**
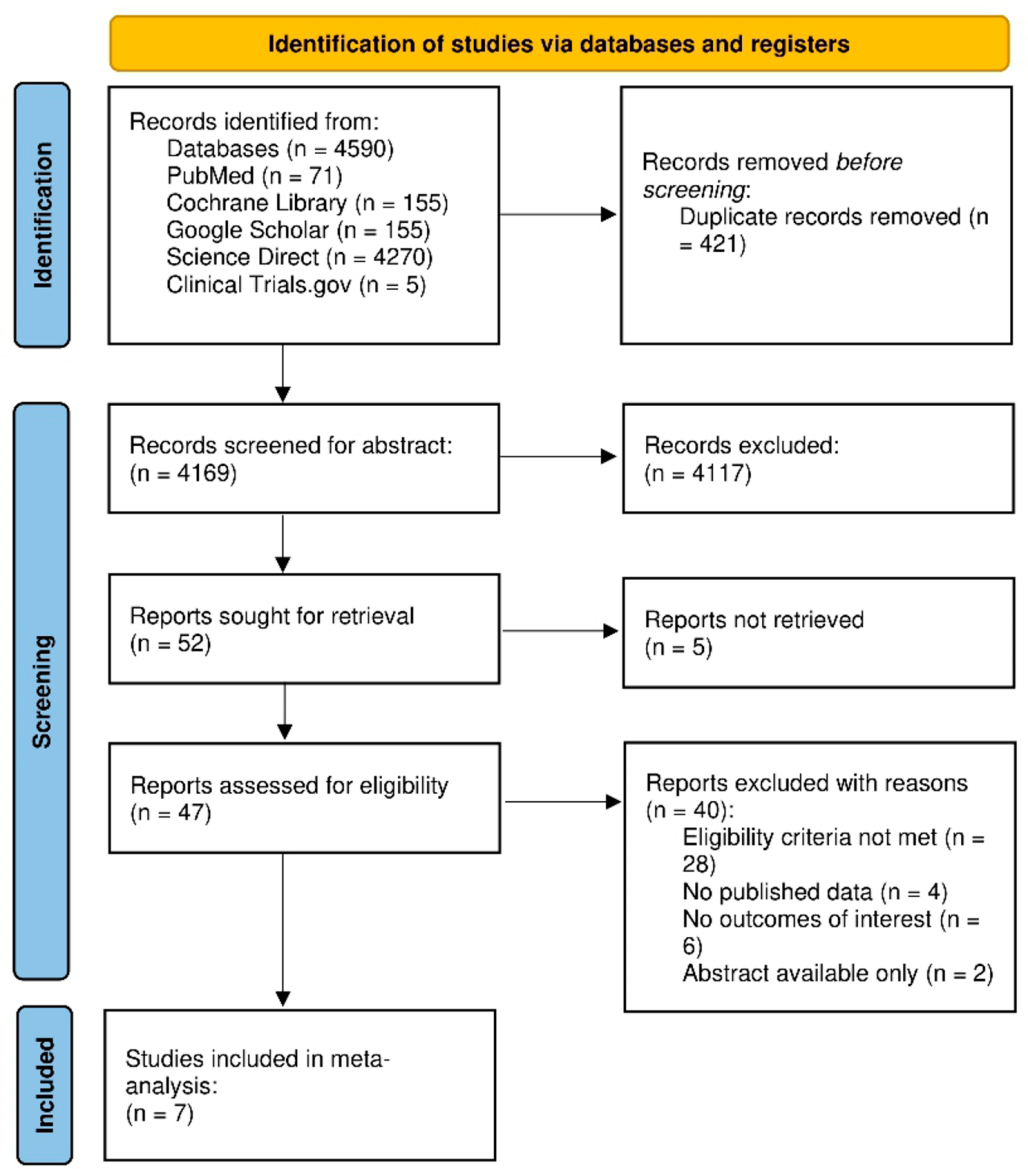
Prisma flow chart of literature search

An initial search across five electronic databases and various other sources yielded 4,590 studies. We yielded 4,169 records after removing duplicates. A thorough screening of titles and abstracts resulted in the exclusion of 4,117 studies. 52 articles were identified for retrieval, although 5 could not be obtained due to the unavailability of full text. Out of the 47 articles subjected to a full-text review for eligibility, 40 were excluded for failing to meet the inclusion criteria. Consequently, 7 RCTs were selected for our meta-analysis [3, 4, 16–20]. The comprehensive literature search process is delineated in the PRISMA flowchart in Fig. 1.

### Studies’ and patients’ baseline characteristics

The current study included 236 participants in the LTAP group and 232 participants in the UTAP group. Including the 135 participants who did not fall into any specific group, the total number of participants was 603 from a total of 7 RCTs. Three of the studies included were conducted in Turkey, while the remaining 4 were from India and Egypt. Mean BMI ranged from 26.5 kg/m² to 29.9 kg/m² with an average BMI of 28.1 kg/m² in the LTAP group. However, in the control group, the range varied from 27.3 kg/m² to 30.7 kg/m² with a mean of 29.13 kg/m². The mean age of participants in the intervention group was 44 years while ranging from 38.1 years to 50 years. In the UTAP group, the mean age of participants was 45 years while the range varied from 39.4 years to 55 years. Tables 1 and 2 extensively cover studies’ and patients’ baseline characteristics.

**Table 1:** Study characteristics

Author & Year	Study Design	Region/ Place of Study	Mean BMI (SD)		Mean operation time (SD)		Outcomes assessed
			LTAP	UTAP	LTAP	UTAP	
Uzunay NT, et al. 2024 [4]	RCT	Turkey	28.33 (5.11)	27.33 (3.46)	46.83 (10.7)	45.03 (8.38)	VAS, PONV, time to first bowel evacuation.
Sahap M, et al., 2023 [20]	RCT	Turkey	26.5 (3.6)	28.2 ± 4.5	NR	NR	VAS.
Soytürk İ, et al. 2023 [3]	RCT	Turkey	27.8 (6.55)	30.3 (4.8)	41 (8.25)	36 (8.5)	VAS, time to first analgesic need, time to first bowel evacuation, time to first flatus.
Emile SH, et al. 2022 [21]	RCT	Egypt	29.9 (6.6)	30.7 (5.8)	63.5 (14.6)	59.5 (9.4)	VAS Score, time to first analgesic need, PONV, time to first flatus.
Fattoh, et al. 2020 [16]	RCT	Egypt	NR	NR	63.55 (12.04)	60.2 (11.47)	VAS, PONV, postoperative morphine consumption.
Venkatraman R, et al. 2020 [19]	RCT	India	NR	NR	50.48 (5.89)	52.91 (6. 62)	VAS, postoperative morphine consumption
Ravichandran NT, et al. 2017 [18]	RCT	India	NR	NR	NR	NR	VAS, time to first analgesic need, postoperative morphine consumption, PONV, time to first bowel evacuation, time to first flatus.

BMI, Body Mass Index; SD, standard deviation, VAS, Visual analog scale; PONV, Postoperative nausea vomiting

**Table 2:** Population characteristics

Author (year)	Total no. of participants (*n*)	LTAP (no. of participants)	UTAP (no. of participants)	Mean age (SD)		Sex
				LTAP	UTAP	
Uzunay NT, et al. 2024 [4]	60	30	30	43.7 (15.54)	48.97 (13.68)	M = 12 (20%) F = 48 (80%)
Sahap M, et al., 2023 [20]	63	21	21	48.0 (8.9)	47.5 (11.8)	M = 20 (47.61%) F = 22 (52.38%)
Soytürk İ, et al. 2023 [3]	170	59	55	50 (14.25)	55 (13.75)	M = 39 (34.21%) F = 75 (65.79%)
Emile SH, et al. 2022 [21]	110	36	36	41.1 (11.8)	40.9 (12.5)	M = 9 (12.5%) F = 63 (87.5%)
Fattoh, et al. 2020 [16]	60	20	20	41.45 (11.20)	42.05 (9.70)	NR
Venkatraman R, et al. 2020 [19]	80	40	40	38.06 (10.06)	39.38 (8.32)	M = 27 (33.75%) F = 53 (66.25%)
Ravichandran NT, et al. 2017 [18]	60	30	30	45.73 (14.44)	41.5 (14.62)	M = 16 (26.6%) F = 44 (73.3%)

M, males; F, females, SD, standard deviation, LTAP, Laparoscopic-assisted transversus abdominis plane block, UTAP, Ultrasound-guided transversus abdominis plane block

### Quality assessment

**Fig. 2:**
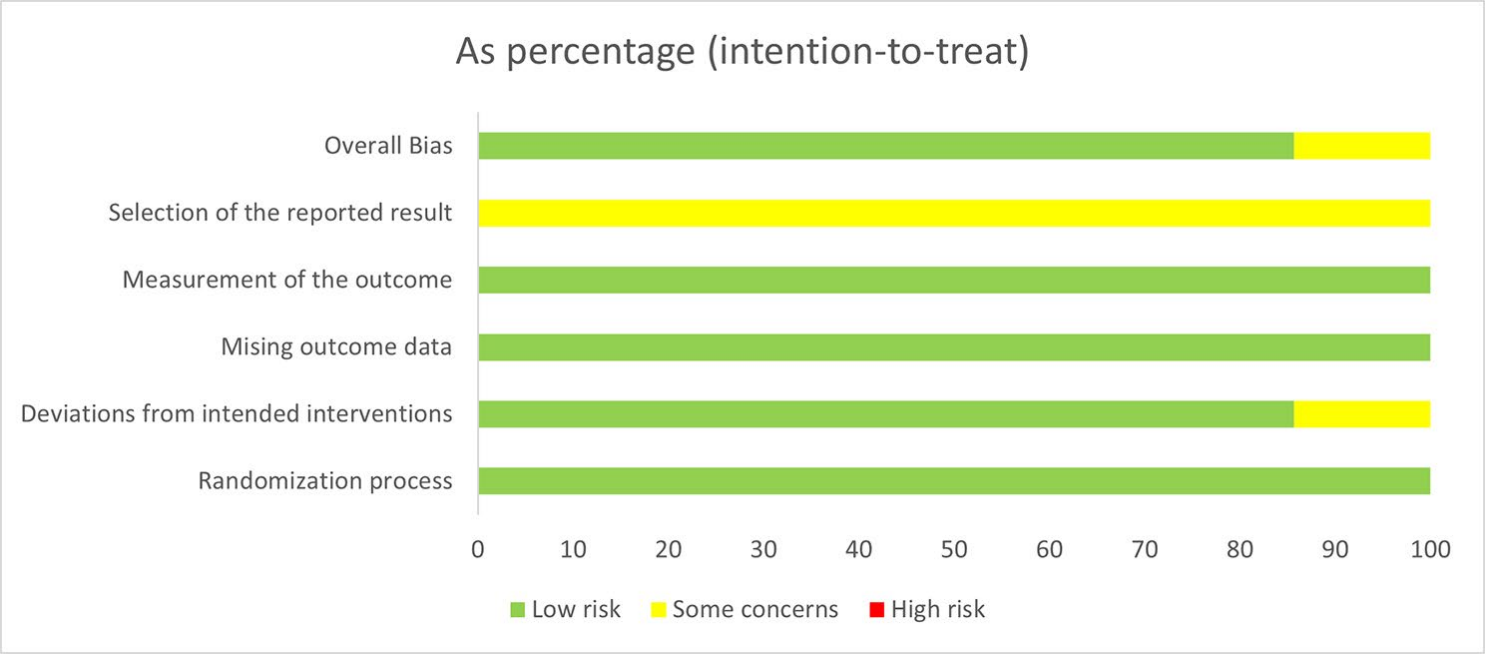
Risk of bias graph

**Fig. 3:**
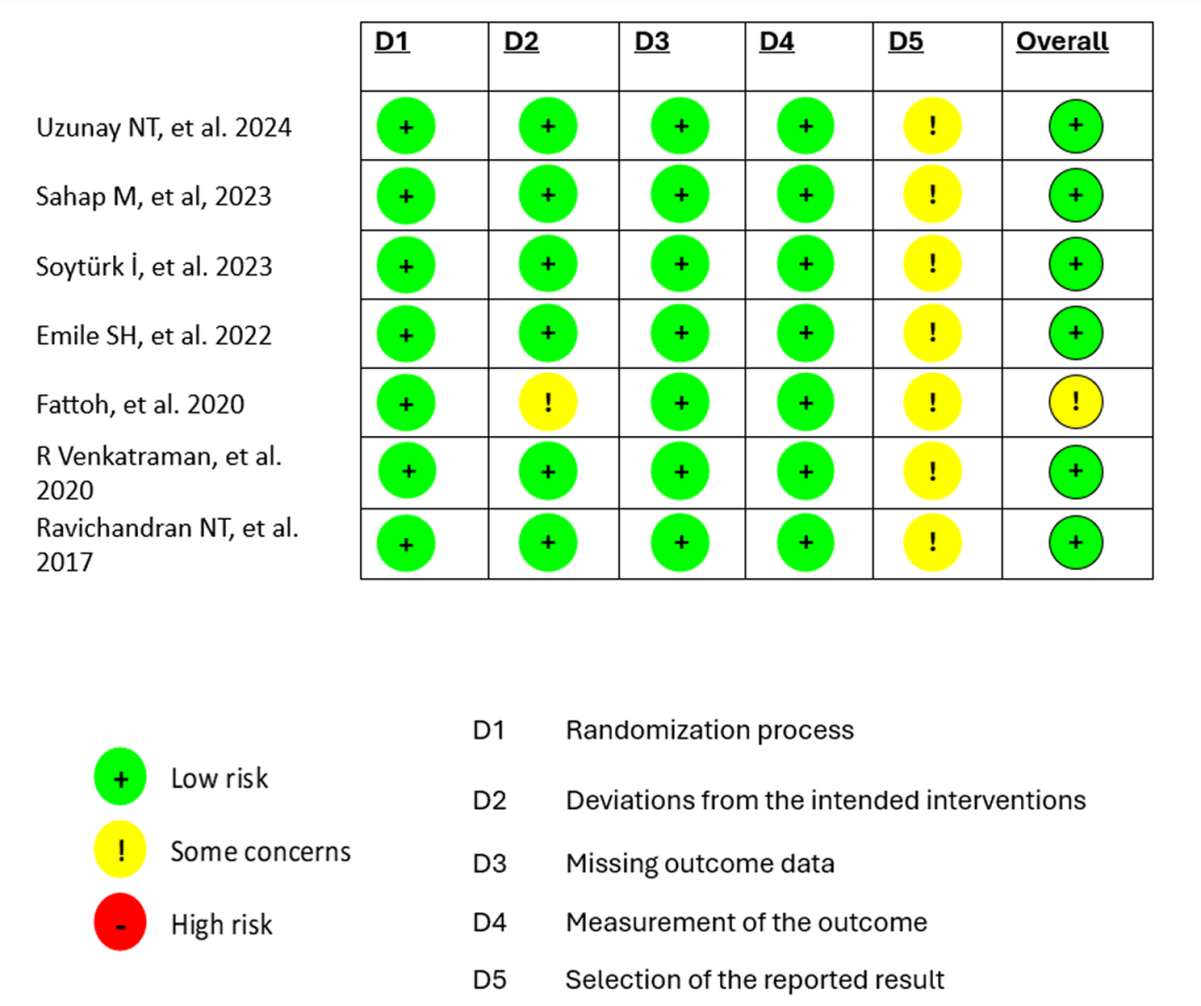
Risk of bias summary

Six of the included studies indicated an overall low risk of bias in all five domains enhancing the credibility of this meta-analysis. A single study by Fattoh et al. [16] reported a moderate risk of bias in a single domain due to deviations from intended interventions, which was the sole significant factor that compromised the quality of this RCT. The detailed results of the quality assessment are listed in Figs. 2 and 3.

### Outcome analysis

#### VAS pain score

All seven studies reported VAS scores for post-operative pain intensity analysis at 24 h, while only five of them reported VAS scores at 6 and 12 h post-operation. Analysis of all studies suggested no statistically significant reduction in postoperative pain in patients receiving LTAP block compared to the UTAP group at 6, 12, and 24-hour post-operation (6 h: WMD = 0.09; 95% CI = -0.47, 0.65; *P* = 0.75; I² = 74%, 12 h: WMD = -0.23; 95% CI = − 0.85, 0.38; *P* = 0.46; I² = 72%, 24 h: WMD = − 0.15; 95% CI = − 0.06, 0.31; *P* = 0.53; I² = 79%) (Supplementary Fig. 1).

**Fig. 4:**
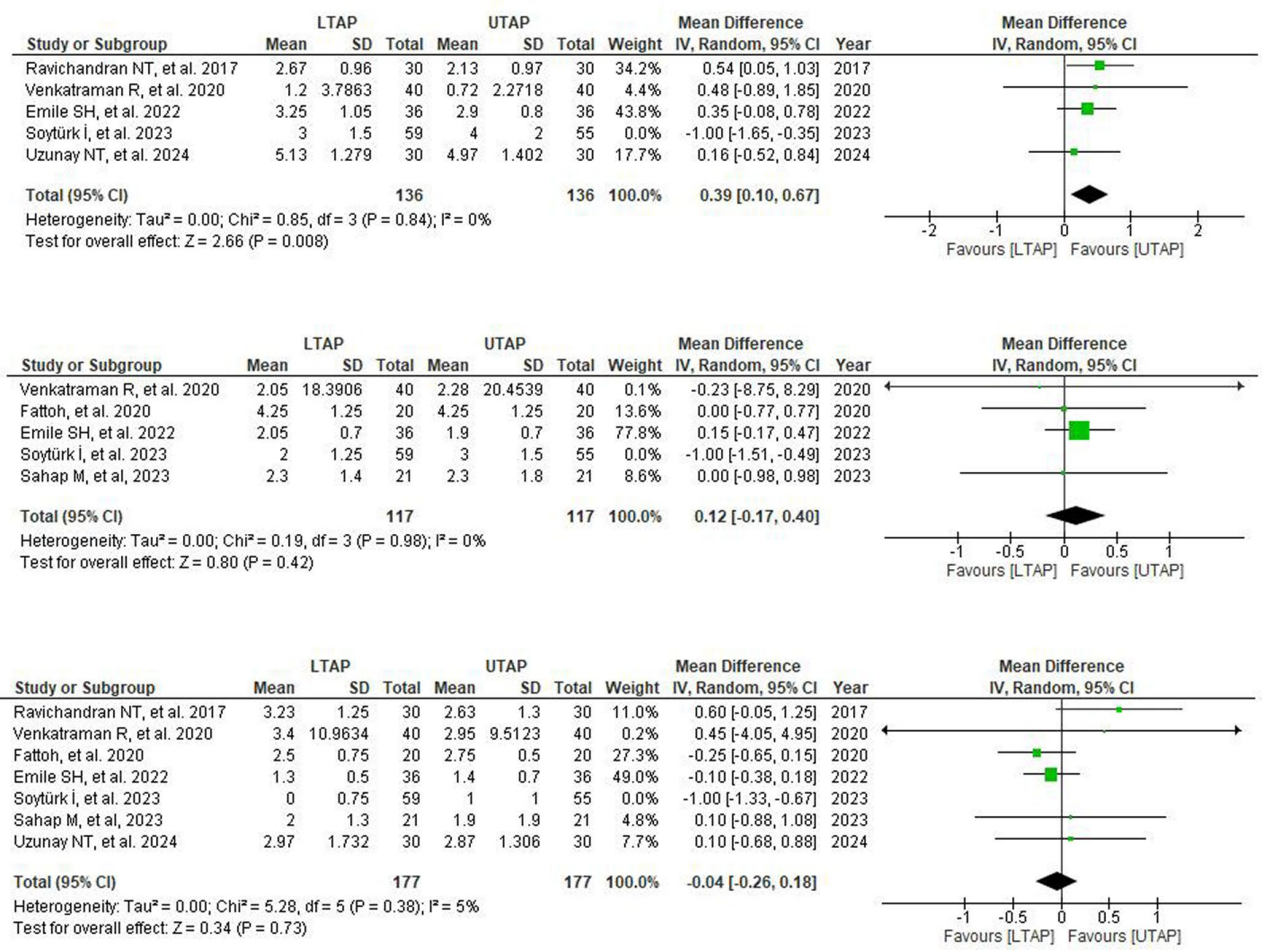
Post-sensitivity analysis forest plot depicting mean differences for VAS scores in LTAP vs. UTAP group at (**A**) 6th hour post-operation (**B**) 12th hour post-operation (**C**) 24th hour post-operation

A moderate level of heterogeneity was observed amongst all studies at 6 and 12-hour time frames while a high level of heterogeneity was observed at 24-hour timeframe. Heterogeneity was markedly reduced by performing sensitivity analysis for each timeframe by removing one study (Soyturk I, 2023) [3]. It showed significantly reduced postoperative pain in patients receiving LTAP relative to UTAP patients at 6-hour post-operation (6 h: WMD = 0.39; 95% CI = 0.10,0.67; *P* = 0.008; I² = 0%) but sensitivity analysis yielded no significant difference at 12 and 24 h (12 h: WMD = 0.12; 95% CI = -0.17,0.40; *P* = 0.42; I² = 0%); 24 h: WMD = -0.04; 95% CI = -0.26, 0.18; *P* = 0.73; I² = 5%) (Fig. 4).

### Time to first analgesic need

**Fig. 5:**
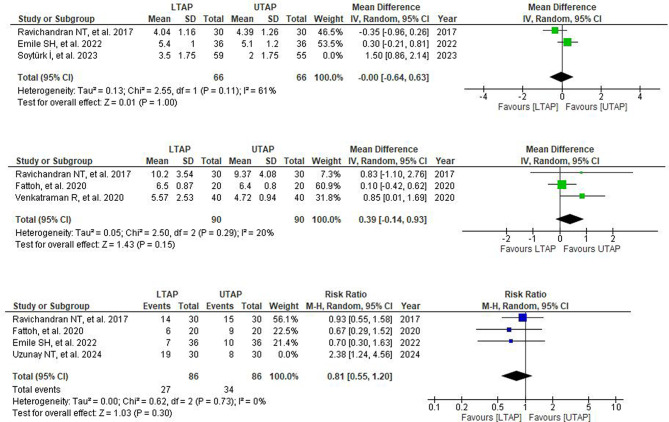
(**A**) Post-sensitivtiy analysis forest plot depicting mean differences for time to first analgesic need (hr) in patients receiving LTAP vs. VTAP group. (**B**) Post-sensitivity analysis forest plot depicting mean differences for Postoperative morphine consumption in patients receiving LTAP vs. VTAP group. (**C**) Post-sensitivtiy analysis forest plot depicting risk ratios for Post-operative nausea and vomiting (PONV) in patients receiving LTAP vs. VTAP group

Three out of seven studies reported time to first analgesic need. No significant difference was observed between the groups (WMD = 0.48; 95% CI = − 0.51,1.47; *P* = 0.35; I^2^ = 88%) (Supplementary Fig. 2). A high level of heterogeneity was observed among the included studies, for which leave one out sensitivity analysis was performed, and heterogeneity was reduced by leaving (Soyturk I et al.) [3]. (WMD = -0.00; 95% CI = − 0.64, 0.63; *P* = 1.00; I^2^ = 61%) (Fig. 5).

### Postoperative morphine consumption

Three out of seven studies reported data on postoperative morphine consumption that showed no statistically meaningful difference between the two groups (WMD = 0.39; 95% CI = − 0.14, 0.93; *P* = 0.15.; I^2^ = 20%) Only a mild level of heterogeneity was observed among the included studies (Fig. 5).

### Postoperative nausea and vomiting (PONV)

Four out of seven studies reported data on postoperative nausea and vomiting. The combined analysis showed no significant difference between the LTAP and UTAP groups (RR = 1.04; 95% CI = 0.59, 1.86; *P* = 0.89; I^2^ = 63%). (Supplementary Fig. 2) A moderate level of heterogeneity was observed among the included studies and on performing sensitivity analysis by removing one study (Uzunay NT, et al.) [4], heterogeneity was markedly reduced but showed no significant difference in the incidence of PONV in LTAP as compared to the UTAP group (RR = 0.81; 95% CI = 0.55, 1.20; *P* = 0.30; I^2^ = 0%) (Fig. 5).

### Time to first bowel evacuation

**Fig. 6:**
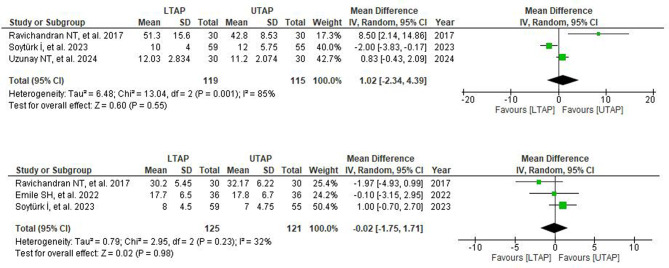
(**A**) Forest plot depicting mean differences for time to first bowel evacuation (hr) in patients receiving LTAP vs. VTAP group. (**B**) Forest plot depicting mean differences for time to first flatus (hr) in patients receiving LTAP vs. VTAP group

Three out of seven studies reported data on the time to passage of the first stools. Following pooled analysis, no substantial difference was observed between the groups (WMD = 1.02; 95% CI = − 2.34, 4.39; *P* = 0.55; I^2^ = 85%). A leave-one-out sensitivity analysis was performed for high heterogeneity, which revealed that no particular RCT contributed to the observed heterogeneity (Fig. 6).

### Time to first flatus

Among the seven studies reviewed, three provided data on time to the first flatus. The combined analysis revealed no notable difference between the LTAP and UTAP groups (WMD = − 0.02; 95% CI = − 1.75, 1.71; *P* = 0.98; I^2^ = 32%) with only mild heterogeneity (Fig. 6).

## Discussion

In this systematic review and meta-analysis, we compared the effectiveness of LTAP against UTAP block in patients undergoing LC, in terms of their post-operative pain reduction, time to first analgesic need, postoperative morphine consumption, PONV, time to first bowel evacuation, and first flatus elimination. Data from seven RCTS, consisting of a total of 603 patients, 236 in the LTAP group and 232 in the UTAP group, were pooled in this systematic review and meta-analysis. Our study findings imply a significant impact of LTAP on the 6-hour postoperative VAS pain score relative to UTAP. However, it showed no significant impact on the 12 and 24-hour postoperative VAS scores and the rest of the outcomes. The large difference in pain relief found at both 6^−^hour post-operative indicates that earlier use of this technique immediately following LC coupled with more aggressive treatment may provide maximum earlier phase patient comfort. This in turn may result in improved ambulation, faster time to hospital discharge, and less dependence on opioids or other analgesic medications with their associated side effects and risks.

This new method has shown similar reductions in pain scores and narcotic usage during postoperative periods [21]. Reduction in pain has been the primary factor driving the evolution of LC into a daycare procedure.

Our findings demonstrated a statistically significant difference in the 6-hour VAS score for postoperative pain in patients receiving LTAP compared to those receiving UTAP blocks. In contrast, Ravichandran et al. reported that patients in the UTAP group had lower VAS scores at 6 and 24 h compared to the LTAP group, though this difference was not statistically significant [17]. Findings in our paper also contradicted Venkatraman et al. who reported that patients in the UTAP group had a lower VAS score at 8 h than those in the LTAP group [18]. Studies comparing UTAP or LTAP blocks with control groups have shown that TAP blocks reduce pain scores. The difference in VAS scores between UTAP and LTAP blocks may be due to the precise needle tip visualization in the UTAP, whereas the LTAP technique is semi-blind, relying on Doyle’s internal bulge sign, which is a bulge seen using the laparoscope when the drug is administered in the correct plane [17]. However, for further clinical relevance in terms of pain assessment, more studies evaluating pain using qualitative measures are needed.

Our analysis has shown no significant difference in time to first analgesic need and postoperative morphine consumption, however, some studies in the literature that compared LTAP with UTAP blocks reported lesser total consumption of morphine in the UTAP block group [17, 18]. 

A very common side effect of opioids is bowel dysfunction and constipation, therefore, one of the parameters in our systematic review and meta-analysis was assessing the time to pass the first stool and time to pass the first flatus elimination between the two groups. Our analysis yielded no statistically significant difference. However, Ravichandran and the team reported that the time taken for the passage of the first stools postoperatively was comparatively longer in the LTAP group and the difference was statistically significant. The time taken for the passage of the first flatus showed no significant difference between the two groups, which was in concordance with the findings of our paper [17]. Similarly, there was also no significant reduction in postoperative nausea and vomiting. Uzunay et al. reported in their study that nausea and vomiting, along with the necessity for antiemetics was lower in the UTAP group but it was not statistically significant [4]. 

There can be several factors attributed to the moderate heterogeneity observed in our meta-analysis. These include variations in surgical techniques that were dependent on the anesthesiologist’s proficiency or the skill levels of the clinicians performing the blocks. This might have contributed to the differences in outcomes like post-op pain and recovery time [3, 4, 16, 17, 20]. Another potential contributor to heterogeneity could be the inconsistencies of patients’ characteristics like age, BMI, and American Society of Anesthesiologists (ASA) scores between studies used in the meta-analysis. For instance, older patients or those with comparatively higher BMI might have experienced slower recovery and higher pain levels compared to healthier younger individuals with low BMI [3, 17, 18]. 

Additionally, variability might have arisen from differences in the type and dosages of anesthetics used. Rupivacaine was utilized in some studies, while in others, different concentrations or even alternative drugs like Bupivacaine were used. Alongside, some studies administered a single shot for TAP block while others employed continuous infusion methods [3, 17, 20]. These discrepancies might have led to variations in pain relief and duration for analgesia, contributing to heterogeneity in the results. Further variations in postoperative management protocol among studies were observed as some used opioids as patient-controlled analgesia and others used fixed doses with NSAIDs or non-opioid analgesics. Patient-controlled analgesia allows for more consistent pain management while fixed doses lead to variability in pain relief experience [3, 17, 18]. 

Finally, while all the included studies were RCTs, there still remained a chance of minor differences between blinding, randomization methods, and sample sizes. All of these variations are important to consider while interpreting the results of our meta-analysis [3, 4, 17]. 

### Strengths and limitations

As of yet, no systematic review and meta-analysis has evaluated LTAP with UTAP in terms of the various post-operative outcomes as reported in our study. Our meta-analysis is the first pooled result of RCTs comparing LTAP with UTAP in improving postoperative outcomes for LC. The randomized, double or single-blinded approach as well as the low risk of bias of the studies included in this comprehensive meta-analysis further strengthens the credibility of the current paper.

However, a few limitations have hindered the authenticity and the acceptability of the results of this study. Firstly, the comparatively moderate sample size and moderate heterogeneity despite sensitivity analyses of the various outcomes in our study may limit the statistical power of the paper.

Secondly, our study included only seven RCTs, which limited us in analyzing publication bias by performing tests like Egger’s, Begg’s, and Rank’s correlation or generating funnel plots as they require a minimum of 10 RCTs. This may reduce the strength and reliability of our conclusions. We acknowledge the limitation and recommend further research with a larger number of studies to provide more generalizable insights.

## Conclusion

In conclusion, the pain reduction, except for the 6th-hour VAS score, post-operative opioid consumption, PONV, time for passing stool, and flatus elimination were statistically similar in both the comparable cohorts. Some large-scale RCTs are required to have more conclusive findings. There is no consensus on which technique has an edge over the other, LTAP, however, is preferred because it does not require additional equipment and an experienced anesthesiologist. Further studies should focus on procedure-specific outcomes.

Further, large-scale RCTs are needed globally to expand research prospects in comparison studies of LTAP with UTAP for LC. Future studies should prioritize procedure-specific outcomes such as long-term pain control and patient recovery patterns while also focusing on cost-effectiveness and evaluating outcomes in specific patient subgroups, to determine whether LTAP or UTAP offers distinct advantages in particular clinical scenarios and populations.

## Electronic Supplementary Material

Below is the link to the electronic supplementary material.

Supplementary Material 1

## Data Availability

The data presented in this meta-analysis are provided in this article/ Supplementary material. Further inquiries can be directed to the corresponding author/s.

## Declarations

### Registration and protocol

The review protocol for this meta-analysis can be accessed through the PROSPERO registry at the following link: https://www.crd.york.ac.uk/prospero/ with the Registration ID: CRD42024551599.

### Amendments to registration or protocol

There have been no amendments to the information provided at registration or in the protocol.
